# Nutritional Composition and Content of Bioactive Compounds in Field Pea and Chickpea Varieties as Functional Raw Material for the Food Supply Chain

**DOI:** 10.1021/acsfoodscitech.5c00941

**Published:** 2025-12-03

**Authors:** Manuel Martoccia, Matteo Donna, Paolo Colombatto, Jean Daniel Coïsson, Fabiano Travaglia, Massimo Blandino

**Affiliations:** † Dipartimento di Scienze del Farmaco, 19050 University of Piemonte Orientale, Largo Donegani 2, Novara, NO 28100, Italy; ‡ Dipartimento di Scienze Agrarie, Forestali e Alimentari, 9314 University of Turin, Largo Braccini 2, Grugliasco, TO 10095, Italy

**Keywords:** pulses, genotypes, amino acids, phenolic compounds, antioxidant activity

## Abstract

Despite the increasing demand for plant-based foods that has renewed interest in pulses as functional ingredients, limited studies have compared the compositional variability among commercial chickpea and field pea cultivars. This study compared the compositional variability of 12 field pea and 11 chickpea cultivars cultivated under identical conditions in Northwest Italy. Comprehensive nutritional and phytochemical profiles, including macronutrients, carotenoids, phenolics, flavonoids, and antioxidant activities, were assessed. Among field peas, the Bluemoon cv showed the highest starch content (57.5 g 100^–1^ g d.w.), supporting its suitability for extrusion-based products, while Angelus exhibited the highest phenolic content (154.2 mg kg^–1^ d.w.) and antioxidant activity. In chickpeas, the desi cv Nero stood out for its elevated dietary fiber (21.5 g 100 g^–1^ d.w.), phenolics (37.2 mg kg^–1^ d.w.), and antioxidant capacity. These findings highlight the cultivar selection value to improve the nutritional and functional properties of pulse-based foods and support sustainable innovation in food-product development.

## Graphical abstract

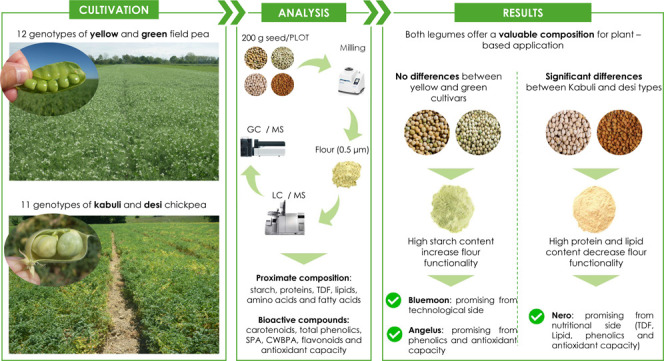

## Introduction

In recent years, consumer demand for sustainable and plant-based foods has grown markedly, driven by dietary trends such as veganism and vegetarianism and by the imperative to reduce environmental impacts.^1,2^ Pulses occupy a central role in addressing these demands: they enrich soils through biological nitrogen fixation, support diversified crop rotations that suppress pests and diseases, and deliver high-quality proteins, dietary fiber, complex carbohydrates, polyunsaturated fatty acids, and micronutrients in the human diet.^3–5^ Among these, field pea and chickpea are particularly relevant because they combine valuable nutritional properties with favorable techno-functional characteristics, making them suitable for bakery, snack, and pasta formulations.^1,6^

Despite these advantages, pulse cultivation in temperate regions has lagged cereals in yield and supply-chain organization.^7,8^ Efforts to revitalize pulse production, through policy incentives, improved crop rotations, and consumer interest in functional foods, have underscored the need for well-characterized raw materials. In particular, plant-based formulations for bakery, snack, and pasta products demand ingredients that not only meet nutritional claims (e.g., high protein, balanced essential amino acids, dietary fiber) but also exhibit reliable techno-functional properties (e.g., starch gelling, protein solubility, and emulsification).^9^ Previous research has characterized individual genotypes of field pea or chickpea for specific traits, such as protein content, starch functionality, or antioxidant levels, but typically focuses on only a handful of cultivars (cvs) or compounds under varied growing conditions.^6,10–13^ As a result, there remains a lack of comprehensive, head-to-head comparisons of multiple commercially relevant genotypes, assessed for both proximate composition and bioactive profiles under uniform agronomic practices.

In the present study, we address this gap by conducting a comprehensive, side-by-side characterization of 12 field pea and 11 chickpea cvs, all grown under identical agronomic and pedoclimatic conditions, assessing proximate composition alongside bioactive compounds in wholemeal flours. Our objective is to identify, within the genotypes more widely cultivated in the considered growing areas, the ones with superior nutritional and functional profiles, thereby guiding seed companies, farmers, and the other operators of the food supply chain in selecting pulse cvs tailored to sustainable, health-oriented food applications.

This study provides the first systematic comparison of nutritional and bioactive profiles across multiple field pea and chickpea cultivars grown under identical agronomic conditions, offering data that can guide targeted breeding and ingredient selection for functional food applications in order to promote a possible transition from a poorly categorized commodity to added-value specialties.

## Materials and Methods

### Experimental Site

Twelve genotypes of field pea and 11 genotypes of chickpea, selected from the conventionally cultivated cvs of each crop in Italy, were considered for the study and cultivated side by side in 2021–22 growing season in the Northwest of Italy, at Polonghera (44°48′N, 7°36′E, altitude 245 m) in sandy-loamy soil. The list of considered genotypes, their main qualitative traits, and the seed company in charge of their commercialization are reported in Table S1. For each field, genotypes were assigned to an experimental unit using a completely randomized block design with three replicates. The plots were measured 7 × 1.5 m. The precipitations and daily temperatures were measured at meteorological stations located near the experimental field (Table S2). The standard agronomic management practices for the area were adopted in all of the plots. The field pea was sown on November 8th and harvested on June 10th, while the chickpea was sown on April 8th and harvested on August 11th. The previous crops were maize and wheat for field pea and chickpea, respectively; in both crops, the soil was plowed (30 cm) and disk harrowed before sowing. Planting was carried out with a plot seeder (model ZÜRN D62se, Woodway, UK) at a seeding rate of 86 and 45 seed m^–2^, for field pea and chickpea, respectively. Weed control was conducted chemically in both crops, both with pre-emergence and postemergence applications. Both crops did not receive any fertilization; according to the growing season, field pea was rainfed, while the chickpea field received one sprinkler irrigation (50 mm) in June.

### Grain Yield and Kernel Quality Traits

Harvesting was carried out using a Walter Wintersteiger (Ried im Innkreis, Austria) cereal plot combine harvester. According to the ordinary crop practice for the growing area, for both crops and all compared cvs, harvest was carried out at the storage moisture content (<12%); thus, no drying process has been applied after harvest. The grain yield was calculated as dry weight on a hectare basis. The harvested grains were mixed thoroughly, and approximately 1 kg grain samples were taken from each plot to determine the grain moisture content and the test weight, which was done using a GAC 2000 Grain Analyzer (Dickey-John Corp., Auburn, IL, USA). The thousand-kernel weight was determined on two 200-kernel sets of each sample using an electronic balance. Representative subsamples (500 g) were ground to wholemeal for proximal and phytochemical analysis using a laboratory centrifugal mill equipped with a 500-μm sieve (Model ZM-200, Retsch, Haan, Germany).

### Chemicals

Water (HPLC grade) was obtained with a MAINA Ultrapure 1760 (G. Maina, Pecetto Torinese, Turin, Italy). Formic acid (99%, LC–MS grade), acetonitrile (ACN, HPLC grade), methanol (HPLC plus gradient), dichloromethane (for analysis ACS Reag. Ph. Eur. and Reag. USP, stabilized with amylene), diethyl ether (for analysis ACS stabilized with BHT), sulfuric acid 96% (for analysis ISO), hydrochloric acid 34–37% (Superpure, for trace analysis), sulfuric acid 0.1 N (for analysis), sodium hydroxide 0.1 N (for analysis), sodium hydroxide pellets (for analysis), and Folin–Ciocalteu’s reagent were purchased from Carlo Erba (Milan, Italy). Megazyme Total Dietary Fiber (TDF) Analysis kit and Celite (acid washed) are from Megazyme (Wicklow, Ireland). Myricetin ≥97%, (±)-catechin ≥96% and (−)-epicatechin ≥97%, orthophosphoric acid 85%, Coomassie Brilliant blue G250, and Supelco 37 component FAME Mix were purchased from Merck Life Science (Milan, Italy); hyperoside ≥98%, apigenin-7*-O-*glucoside ≥99%, rutin ≥99%, and quercitrin ≥98.5% were purchased from Extrasynthèse (Genay, France). 2-2′-Azino-*bis*(3-ethylbenzothiazoline-6-sulfonic acid) diammonium salt (ABTS), ethanol (CHROMASOLV, 99.8%), ethyl acetate (CHROMASOLV, 99.8%), (±)-6-hydroxy-2,5,7,8-tetramethylchromane-2-carboxylic acid (Trolox, 97%), hydrochloric acid (HCl, 37.0%), iron­(III) chloride hexahydrate (FeCl_3_·6H_2_O, ≥98.0%), methanol (CHROMASOLV, 99.9%), potassium sulfate (K_2_SO_4_, ≥99.0%), sodium hydroxide (NaOH, ≥98.0%), 2,4,6-Tris­(2-pyridyl)-s-triazine (TPTZ), and phenolic acid standards (caffeic acid ≥98%, *p*-coumaric acid ≥98%, *t*-ferulic acid ≥99%, gallic acid ≥99%, protocatechuic acid ≥99%, *p*-hydroxybenzoic acid ≥99%, sinapic acid ≥98%, syringic acid ≥95%, and vanillic acid ≥ 97%) were purchased from Sigma-Aldrich (St. Louis, Missouri, USA).

### Proximate Composition Analysis

The starch content was measured according to the Official Journal of the European Communities standard method (L 209/25, 1999).^14^ The total protein content was obtained according to the Kjeldahl method (conversion factor: 6.25) with the Kjeltec system I (FOSS Italia S.p.A., Padova, Italy).^15^ The TDF assay was performed according to the AOAC 991.43 enzymatic–gravimetric method.^16^ The lipid content was determined by the Soxhlet method (6 h of extraction) with a semiautomatic Solvent Extractor System B-811 (Buchi, Switzerland) using dichloromethane as the solvent.^17^ The ash content was determined in a muffle furnace at 525 ± 25 °C. The moisture content was determined using a Sartorius MA30 thermobalance (Sartorius AG, Goettingen, Germany).

### Quantification of the Protein Fractions

A sequential extraction was applied on 50 mg of defatted flour with demineralized H_2_O, NaCl 0.5 N, NaOH 1 N, and EtOH 70% v/v for the extraction of albumins, globulins, glutelins, and prolamins, respectively. For the protein quantification in the extracts, the Bradford assay was applied. Results were expressed as mg bovine serum albumin equivalents (BSAE) protein g^–1^ of dry weight.^18^

### Amino Acids Composition

The quantification of the amino acid profile was done, on a merged representative sample for each cv, by CHELAB s.r.l. (Mérieux NutriSciences, Resana, TV, Italy) using the HPLC–FLD method for the total tryptophan content (AOAC 2017.03) and the HPLC–UV method (MP 2455 rev 1 2022) for the other amino acids. The analysis was done with three different hydrolysis on the matrices for the optimal amino acid’s recovery. Results were expressed as g 100 g^–1^ of dry sample.

### Fatty Acid Methyl Ester (FAME) Analysis

The lipid fraction collected from Soxhlet extraction was transformed into respective fatty acid methyl ester (FAME). Briefly, 200 mg of lipid was treated, in a vial, with sodium methylate 0.5 M in methanol, left to react in a Thermomixer comfort (80 °C, 350 rpm, 10 min) (Eppendorf, Germany), and cooled at room temperature. Then, 200 μL of distilled water and 500 μL of diethyl ether were added; the vials were shaken and left to stand to allow the layers to separate. 50 μL of the diethyl ether containing the FAME phase was transferred into a vial with 950 μL of dichloromethane. The vials were stored at −20 °C until they were analyzed. The FAME analysis was conducted using a Thermo Trace 1300 (Thermo Finnigan, Rodano, Milan, Italy) equipped with a flame ionization detector (FID); a split–splitless injector was used. A DB23 (30 m, 0,25 mm id; 0.25 μm film thickness) (J&W Scientific, Folsom, CA) capillary column coated with (50% cyanopropyl)-methylpolysiloxane stationary phase was used. The injector was operated at 250 °C and the detector at 350 °C. The oven temperature was programmed from 60 to 180 °C (5 °C min^–1^). Hydrogen was used as the carrier gas at 1.5 mL min^–1^; 1 μL of each sample was injected in the split mode (50:1). All the compounds were identified by comparing the retention times (Rt) of the standard mix. The results were expressed as relative percentages.

### Bioactive Compounds and Phytochemicals

#### Carotenoid Determination

Carotenoid content was quantified according to Baleggia et al.’s (2010)^19^ method. Briefly, 0.2 g of flour was collected in a 2 mL plastic tube, and 2 mL of water-saturated butanol was added. The extraction was conducted in an ultrasonic bath (Branson 1510) for 15 min under dim light conditions; after centrifugation (20800 g, 5 min), the solution’s absorbance was detected with the spectrophotometer (Shimadzu UV-1900, Milan, Italy) at 435.8 nm. The results were expressed as mg of β-carotene equivalent 100 g^–1^ of d.w.^19^

#### Total Phenolic Content (TPC)

The defatted flour from the Soxhlet extraction was used for total phenolic quantification. For the extraction, 0.3 g of defatted flour was suspended in 1 mL of MeOH/H_2_O 80:20 (v/v) and mixed by vortexing for 30 s; the extraction was performed in an ultrasonic bath for 30 min. The sample was centrifuged (5 min, 20800 g, 4 °C), and the supernatant was separated. The same procedure was repeated twice on the residue. The Total Phenolic Content (TPC) was determined using a modified Folin–Ciocalteu method.^20^ Briefly, 50 μL of Folin–Ciocalteu reagent and 175 μL of aqueous Na_2_CO_3_ (5 wt %) were added to 30 μL of polyphenolic extract. The solution was then diluted to a final volume of 2900 μL with distilled water. The absorbance was measured spectrophotometrically at 760 nm after storing for 1 h in dark conditions. The results were expressed as mg catechin equivalents (CAE) per g d.w.^21^

#### Soluble (SPA) and Cell-Wall Bound Phenolic Acid (CWBPA)

The extraction and quantification of the SPAs (free and conjugated) and CWBPAs were performed according to Giordano et al. (2019).^22^ The phenolic extracts were filtered through a 0.2 μm filter and then analyzed by means of an Agilent 1200 Series (Agilent Technologies, Santa Clara, CA, USA) high-performance liquid chromatograph coupled with an Agilent 1200 Series diode array detector. Separations were carried out using a 150 mm × 4.6 mm, 5 μm particle size Gemini RP-18 column 129 (Phenomenex, Torrance, CA, USA).

#### Total Flavonoid Content (TFC) and Profile

Flavonoids were extracted according to a modified version of Zhang et al.’s (2015)^23^ method. Briefly, 2.5 g of defatted flour was suspended in 10 mL of an ethanol/water 70:30 (v/v) solution brought at pH 4 with 2 N HCl. The suspension was homogenized by vortexing, and the extraction was performed with an ultrasonic bath (Branson 1510) at room temperature. The mixture was stored at −20 °C for 10 min and centrifuged (5 min, 20800 g, 4 °C), and the supernatant was filtered using a 0.22 μm syringe filter. The filtrates were stored at 4 °C until they were analyzed.^23^ Flavonoid quantification was performed using a Shimadzu LC-20A Prominence chromatographic system equipped with a diode array detector (DAD detector SPD-M20A) (Shimadzu Italia, Milan, Italy). A security guard column containing a C18 phase and a reversed-phase Luna C18 (2), 100 Å LC column (150 × 2.0 mm inner diameter, particle size of 5 μm) (Phenomenex, Torrance, CA, USA) was used at 30 °C. Eluent A was water/formic acid 0.1% v/v and Eluent B ACN/formic acid 0.1% v/v. The elution program was from 5% to 35% B (40 min), from 35% to 75% B (3 min), isocratic 75% B (10 min), from 75% to 5% B (7 min), and isocratic 5% B to equilibrate the column (20 min). The flow rate was 0.4 mL min^–1^, and the total run time was 80 min. DAD detection was performed at 280 and 330 nm. The injection volume was 5 μL. Data acquisition was performed by means of LC Solutions (ver. 1.25, Shimadzu Italy). The single flavonoids were identified by comparing the retention times (Rt) and the UV–vis spectra with those of authentic standards; quantification was performed based on calibration curves obtained with the same standards; the results were expressed as mg kg^–1^ d.w.^24^

#### Antioxidant Capacity (AC)

The total AC was determined by means of FRAP (ferric reducing antioxidant power) and ABTS [2,2’-azino-*bis*(3-ethylbenzthiazoline-6-sulfonic acid)] assays adapted from the QUENCHER method, as described by Giordano et al. (2019).^22^ The results were expressed, through calibration curves, as mmol Trolox equivalents kg^–1^ of sample (d.w.).

#### Statistical Analysis

Results were expressed as an average of at least three independent determinations. The statistical analysis was done with *R* Statistical Software (v4.1.2; R Core Team 2021) and GraphPad Prism version 10 (GraphPad Software, San Diego, California, USA). For each crop, data were first tested for normality using the Shapiro–Wilk test (α = 0.05) to verify parametric assumptions. When data met normality and homogeneity criteria, differences among genotypes were assessed by one-way analysis of variance (ANOVA) followed by Tukey’s multiple comparison test. In cases where the data deviated from a normal distribution, the nonparametric Kruskal–Wallis test was applied, followed by Dunn’s post hoc test for pairwise comparisons. Statistical significance was set at *p* < 0.05 for all analyses. A principal component analysis (PCA) and hierarchical clustering on principal components (HCPC) were applied separately for each crop for a better comprehensive analysis between cvs considering bioactive compounds and antioxidant activity as variables.

## Results and Discussion

### Parametricity Tests

Normality of the data was verified by the Shapiro–Wilk test (α = 0.05). Depending on the outcome, either one-way ANOVA followed by Tukey’s multiple comparison test or the nonparametric Kruskal–Wallis test coupled with Dunn’s post hoc test was applied to assess genotypic differences. The complete results of the Shapiro–Wilk test are reported in Table S3.

### Agronomic Traits

The medium field pea yield was 3.5 t ha^–1^, with no difference between green or yellow genotypes, while the chickpea yield reached on average 0.8 t ha^–1^ (Table 1). Reported yield potentials were in line with the agronomic performance of these crops in the temperate growing areas.^25,26^ Among the considered genotypes, chickpea showed a higher variability between varieties for grain yield (coefficient of variation, CV of 38%) than field pea (CV = 18%), while the variability in terms of thousand kernel weight (TKW) and test weight (TW) between the genotypes was similar. Nero exhibited a lower TKW compared to the kabuli genotypes, apart from Flamenco, which showed similar values, whereas the TW was the second-highest value after Pascià.

**Table 1:** Grain Yield, Thousand Kernel Weight (TKW), and Test Weight (TW) of Cultivars of Field Pea and Chickpea Cultivated in North Italy in 2021–22 Growing Season^a^

crop	qualitative trait	cultivar	grain yield (t ha^–1^)	TKW (g)	TW (kg hL^–1^)
field pea	green	Standal	2.3 A	241 A	88.0 ab
		Verbal	3.7 A	209 A	87.8 ab
		Faquir	3.9 A	202 A	85.7 bcde
		LS Envergure	2.7 A	189 A	87.3 abcd
		Paddle	4.3 A	158 A	84.5 e
		Aviron	4.1 A	155 A	84.7 de
		Bluemoon	3.4 A	150 A	86.7 abcde
	yellow	Astronaute	2.9 A	226 A	88.8 a
		Navarro	2.9 A	221 A	88.0 ab
		Angelus	4.1 A	211 A	86.3 abcde
		RGT Lapony	4.1 A	179 A	85.0 cde
		Enduro	3.3 A	150 A	87.4 abc
					
chickpea	kabuli type, beige	Alamo	1.2 a	449 a	77.5 g
		Pascià	0.7 ab	378 b	82.5 a
		Gavdos	0.3 b	362 c	79.9 cde
		Lambada	1.0 a	355 c	79.0 ef
		APSC4	0.6 ab	349 c	78.3 fg
		APSC3	0.6 ab	332 d	78.3 fg
		Cicerone	1.0 a	318 de	80.4 bcd
		Vulcano	0.4 b	306 e	79.1 def
		Sultano	0.6 ab	304 e	80.9 bc
		Flamenco	0.9 a	269 f	80.5 bc
	desi type, black	Nero	1.0 a	261 f	81.6 ab

a All data are expressed as the average of 3 replications. Within each crop, different lowercase letters indicate significant differences among genotypes according to ANOVA and Tukey’s multiple comparison test. Capital letters denote significant differences identified by Dunn’s post hoc test applied to nonparametric data (following Kruskal–Wallis test).

### Proximate Composition Analysis

The proximate compositions of the compared field pea and chickpea are reported in Table 2.

**Table 2:** Proximate Composition of Field Pea and Chickpea Cultivars^a^ ^b^

crop	qualitative trait	cultivar	moisture	starch	total dietary fiber	lipid	protein	ash
field pea	green	Standal	11.5 A	47.9 AC	16.9 a	2.46 abc	22.8 bc	3.07 a
		Verbal	11.1 A	38.3 C	19.8 a	2.13 c	21.6 ef	3.02 a
		Faquir	8.68 A	49.6 AC	15.9 a	2.46 abc	23.7 a	3.16 a
		LS Envergure	9.43 A	48.4 AC	18.7 a	2.55 abc	21.4 f	3.27 a
		Paddle	11.6 A	51.9 AB	18.8 a	2.55 abc	22.4 bcd	3.14 a
		Aviron	11.3 A	46.1 AC	19.3 a	2.47 abc	22.7 bcd	3.09 a
		Bluemoon	11.8 A	57.5 A	19.3 a	2.65 ab	22.9 b	3.22 a
	yellow	Astronaute	9.49 A	49.8 AC	17.1 a	2.17 c	22.9 b	2.97 a
		Navarro	9.53 A	49.5 AC	16.9 a	2.44 abc	22.1 cde	3.19 a
		Angelus	8.77 A	49.2 AC	17.4 a	2.23 bc	22.6 bcd	3.17 a
		RGT Lapony	9.94 A	44.8 BC	17.9 a	2.65 ab	21.9 def	3.33 a
		Enduro	9.28 A	51.3 AC	17.6 a	2.71 a	22.1 cde	2.86 a
								
chickpea	kabuli type, beige	Alamo	8.38 ab	41.5 AC	15.9 b	7.41 ab	24.3 ab	3.99 a
		Pascià	7.28 c	41.6 AC	14.8 b	7.54 ab	24.3 ab	3.84 a
		Gavdos	7.50 c	42.2 AB	14.8 b	7.64 ab	24.8 ab	3.93 a
		Lambada	7.57 c	39.3 AC	14.3 b	6.70 ab	19.2 b	3.90 a
		APSC4	7.74 bc	42.3 A	14.3 b	8.44 a	24.2 ab	3.98 a
		APSC3	7.65 bc	41.0 AC	14.0 b	8.61 a	25.5 a	3.88 a
		Cicerone	8.56 a	35.3 C	15.6 b	7.98 ab	24.4 ab	3.91 a
		Vulcano	7.43 c	38.7 AC	15.9 b	8.14 ab	26.3 a	3.98 a
		Sultano	7.78 bc	39.6 AC	15.3 b	7.28 ab	28.0 a	3.98 a
		Flamenco	7.70 bc	41.9 AC	15.0 b	8.73 a	23.6 ab	3.73 a
	desi type, black	Nero	7.58 c	38.0 BC	21.5 a	5.72 b	25.0 ab	4.40 a

a All data are expressed as the average of 3 replications. Within each crop, different lowercase letters indicate significant differences among genotypes according to ANOVA and Tukey’s multiple comparison test. Capital letters denote significant differences identified by Dunn’s post hoc test applied to nonparametric data (following Kruskal–Wallis test).

b Values are expressed in g 100 g^–1^ of dry weight.

Overall, field pea exhibited a higher starch content (average 48.7% d.w.; range 38.3–57.5%) than chickpea (average 40.0% d.w.), with Bluemoon showing the highest value and Verbal the lowest. Chickpea starch content varied similarly, with Cicerone and APSC4 representing the extremes with 35.3% and 42.3% d.w., respectively. Higher starch levels in field peas are consistent with their reported superior expansion properties during extrusion compared to chickpeas, which have higher protein and lipid contents that can reduce puffing and increase hardness.^27,28^ TDF averaged 18.0% d.w. in field peas and 15.6% d.w. in chickpeas, with desi types exhibiting approximately 28% more fiber than kabuli types (15.0% d.w.). Wholegrain flour of both crops meets the EU criteria for “high fiber” labeling (≥6 g 100 g^–1^), making them nutritionally optimal due to their beneficial effects on the intestinal microbiota.^29,30^ However, it should be noted that higher fiber levels can reduce expansion during extrusion by limiting starch gelatinization.^28^

The lipid content was significantly higher in chickpeas (7.6% d.w.; 5.7–8.6% d.w.) than in field peas (2.5% d.w.; 2.1–2.7% d.w.), which is advantageous for oxidative stability and extrusion performance, as lipids can form amylose–lipid complexes that hinder pasting.^9,31,32^ Despite poor differentiation among the field pea cvs, the Nero cv was notable for its low lipid content of 5.7% d.w., in comparison to the kabuli cvs. In contrast, the Flamenco, APCS3, and APCS4 cvs exhibited the highest concentrations (8.7%, 8.6%, and 8.4% d.w., respectively).

Protein content averaged 22.4% d.w. in field peas and 24.5% d.w. in chickpeas, with minimal variation among field pea cvs and greater variability in chickpeas (19.2–28.0% d.w.). Wholegrain of both crops qualifies as “high protein” ingredients according to EU regulations (>20% energy from protein), making them suitable for plant-based formulations.^30^ Nevertheless, in addition to the genetic role, other external factors, including agronomic practices and meteorological trends, can exert a slight influence on this parameter.^33^ Ash content averaged 3.1% d.w. in field peas and 3.9% d.w. in chickpeas, with no significant genotypic differences.

### Protein Quality and Amino Acids Profile

The different protein fractions, quantified by a Bradford assay, are reported in Table 3. In terms of concentration, albumins proved to be the most representative among the four groups, followed by glutelins, globulins, and prolamins for field pea and glutelins, globulins, and prolamins for chickpea. On average, field pea cvs exhibited a higher content of albumins (77.4% d.w.) compared to chickpea cvs (63.8% d.w.). Conversely, chickpeas showed a greater abundance of globulins (20.0% d.w.) and glutelins (14.6% d.w.) than field peas. The distribution of protein fractions was generally consistent between yellow and green field pea cvs, with minor exceptions. The mean value of albumin fraction in the field pea samples was 131 mg BSAE g^–1^ d.w., while Dunn’s test showed no significant differences in the field pea globulin content. Desi cv showed a lower albumin content (89.3 mg BSAE g^–1^ d.w.) and comparatively higher globulin (62.7 mg BSAE g^–1^ d.w.) and glutelin contents (31.6 mg BSAE g^–1^ d.w.) compared to the other kabuli cvs. Prolamins, the least abundant fraction in both species, were present at an average concentration of 3.92 and 3.30 mg BSAE g^–1^ d.w. in field peas and chickpeas, respectively.^6,34^

**Table 3:** Protein Fractions of Field Pea and Chickpea Cultivars^a^ ^b^

crop	qualitative trait	cultivar	albumins	globulins	glutelins	prolamins
field pea	green	Standal	132 abc	13.3 A	2.57 de	25.7 de
		Verbal	156 a	9.72 A	18.3 ab	2.77 de
		Faquir	129 abc	11.5 A	15.0 b	4.43 bcd
		LS Envergure	122 abc	13.1 A	21.8 a	3.24 cde
		Paddle	154 ab	14.7 A	18.4 ab	5.87 ab
		Aviron	121 abc	28.1 A	14.1 b	5.00 bcd
		Bluemoon	115 abc	23.2 A	19.9 ab	3.65 cde
	yellow	Astronaute	148 abc	22.5 A	15.1 b	2.03 e
		Navarro	129 abc	10.7 A	15.5 ab	2.00 e
		Angelus	113 bc	14.0 A	20.1 ab	7.63 a
		RGT Lapony	141 abc	11.0 A	19.3 ab	3.54 cde
		Enduro	110 c	23.5 A	18.0 ab	4.87 bc
						
chickpea	kabuli type, beige	Alamo	132 abc	34.2 cde	27.6 c	7.09 a
		Pascià	118 bcd	58.1 ab	24.9 c	5.12 ab
		Gavdos	138 abc	20.7 e	25.8 c	1.91 d
		Lambada	163 a	45.8 bc	29.8 bc	3.01 bcd
		APSC4	126 bc	28.6 de	31.0 bc	2.19 cd
		APSC3	112 cd	26.1 e	40.8 a	4.49 b
		Cicerone	143 abc	40.4 cd	27.4 c	1.37 d
		Vulcano	143 abc	46.6 bc	32.0 bc	1.13 d
		Sultano	141 abc	65.3 a	26.1 c	4.21 bc
		Flamenco	153 ab	30.2 de	36.4 ab	4.45 b
	desi type, black	Nero	89.3 d	62.7 a	31.6 bc	1.32 d

a All data are expressed as the average of 3 replicates. Within each crop, different lowercase letters indicate significant differences among genotypes according to ANOVA and Tukey’s multiple comparison test. Capital letters denote significant differences identified by Dunn’s post hoc test applied to nonparametric data (following Kruskal–Wallis test).

b Values are expressed in mg bovine serum albumin equivalents (BSAE) protein/g of dry weight (Bradford, 1976).^18^

The highest total amino acid content was exhibited by chickpeas (24.2 g 100 g^–1^ d.w.), while field pea exhibited a TAA of 20.8 g 100 g^–1^ d.w. (Figures S1 and S2). In agreement with previous findings by Kan et al. (2018),^35^ glutamic acid (Glx) was the predominant nonessential amino acid (NEAA) in both species, with concentrations of 3.7 g 100 g^–1^ d.w. in field pea and 4.1 g 100 g^–1^ d.w. in chickpea. This was followed by aspartic acid and arginine in both crops. Regarding essential amino acids (EAAs), total contents were 7.82 and 9.34 g 100 g^–1^ d.w. for field pea and chickpea, respectively. Lysine (Lys), leucine (Leu), and phenylalanine (Phe) were the most representative EAAs in both crops. Notable differences were observed in the arginine content, with chickpea showing higher levels (2.4 g 100 g^–1^ d.w.) compared to field pea (1.7 g 100 g^–1^ d.w.). Isoleucine (Ile) was below the limit of quantification (LoQ = 0.010 g 100 g^–1^ f.w.) in Angelus field pea cv, whereas mean values for field pea and chickpea were 0.9 and 1.1 g 100 g ^–1^ d.w., respectively. No remarkable difference was noted between the desi and kabuli chickpea varieties. Both crops are relatively deficient in methionine, tryptophan, and cystine and are rich in lysine.^36^ From a nutritional perspective, the high proportion of EAAs, representing approximately 40% of total amino acids, confirms the good protein quality of these wholegrain flours, contributing meaningfully to dietary protein requirements. EAAs play an important role in muscle protein synthesis, cognitive function, and immune system regulation. Moreover, the degradation of amino acids during processing and storage can lead to the formation of off-flavor compounds. For instance, 2-methylbutanal and 3-methylbutanal, derived from leucine or isoleucine, while methionine can generate sulfur-containing compounds. Pyrazines may originate from alanine, leucine, serine, and threonine.^37^ Based on the amino acid profiles observed, both crops possess comparable potential to generate these volatile compounds during degradation. However, off-flavor formation has been more extensively documented in field pea, while literature on chickpea-associated off-notes remains limited.^38^

### Fatty Acids Profile

The fatty acid compositions of field pea and chickpea genotypes are reported in Table 4, expressed as mean percentages for each class. Saturated fatty acids (SFAs) including palmitic acid (C16:0), stearic acid (C18:0), and arachidic acid (C20:0); monounsaturated fatty acids (MUFAs) such as palmitoleic acid (C16:1), oleic acid (C18:1ω9cis), and eicosenoic acid (C20:1); and polyunsaturated fatty acids (PUFAs) including linoleic acid (LA, C18:2ω6cis) and α-linolenic acid (ALA, C18:3ω3) were identified in all the samples. Linoleic, oleic, and α-linolenic acids were the most representative UFA, while palmitic is the most abundant SFA in both crops.^37,39^ On average, chickpeas exhibited higher oleic acid content (35.1%) than field peas (26.6%), whereas field peas contained significantly more ALA (9.3% vs 2.3%). Field peas also showed a stearic acid content nearly double that of chickpeas (4.0% vs 2.1%). Among field pea types, yellow cvs had higher linoleic acid levels (50.1%) than green ones (46.7%), while green varieties had higher oleic acid content (27.9% vs 24.8%). In chickpeas, the main fatty acids were linoleic acid (48.8%) and oleic acid (35.1%) in the unsaturated fraction, while palmitic acid (10.1%) was the principal saturated acid. Dunn’s test revealed that the Alamo cv stood out due to its stearic acid content (6.8%), significantly above the average (1.6%). The fatty acid profiles showed only minor differences between field peas and chickpeas in terms of quality and quantity. The predominance of unsaturated fatty acids supports their nutritional value; however, high levels of polyunsaturated fatty acids (PUFAs) may lead to the formation of off-flavor compounds, such as hexanal and octanal, from linoleic and oleic acids, respectively.^37^ Maintaining a balanced ω-6/ω-3 ratio is important for cardiovascular health, although no official dietary reference value has been established by EFSA.^40^ Field pea cvs displayed a more favorable ratio (average 5:1), whereas chickpeas showed an unfavorable ratio of 20:1.^41^

**Table 4:** Relative Percentage of the Single Fatty Acids of Field Pea and Chickpea Cultivars^a^ ^b^

crop	qualitative trait	cultivar	C16:0	C16:1	C18:0	C18:1n9cis	C18:2n6cis	C18:3n3	C20:0	C20:1
field pea	green	Standal	10.7 AC	0.07 a	4.29 b	28.4 c	45.1 h	10.3 a	0.61 ab	0.42 ab
		Verbal	9.96 BC	0.05 bc	3.89 de	29.9 b	45.9 g	9.34 bc	0.52 def	0.42 ab
		Faquir	10.2 AC	0.06 abc	4.11 c	25.8 fg	48.7 e	10.2 a	0.55 cd	0.39 ab
		LS Envergure	10.2 AC	0.06 abc	4.10 c	28.4 c	46.1 g	10.1 a	0.58 bc	0.43 ab
		Paddle	10.9 AC	0.05 c	4.37 ab	30.5 a	43.8 i	9.41 bc	0.58 bc	0.40 ab
		Aviron	11.4 AC	0.07 a	3.71 f	26.4 e	49.4 d	8.26 f	0.48 f	0.37 b
		Bluemoon	11.5 AC	0.07 ab	3.80 ef	26.8 de	48.8 e	8.24 f	0.49 ef	0.34 ab
	yellow	Astronaute	10.2 AC	0.07 a	3.91 de	22.7 h	52.7 a	9.49 b	0.54 cde	0.37 ab
		Navarro	10.3 AC	0.07 a	4.02 cd	25.4 g	50.1 c	9.11 d	0.56 bcd	0.40 ab
		Angelus	9.68 C	0.06 ab	3.72 f	27.1 d	49.3 d	9.18 cd	0.48 f	0.47 a
		RGT Lapony	12.1 A	0.07 ab	3.34 g	25.9 f	47.9 f	9.54 b	0.61 ab	0.40 ab
		Enduro	11.9 AB	0.06 ab	4.51 a	22.9 h	50.8 b	8.74 e	0.65 a	0.37 ab
										
chickpea	kabuli type, beige	Alamo	9.26 d	0.13 B	6.77 A	31.7 BC	47.7 AB	2.39 AC	1.74 A	0.34 A
		Pascià	9.83 c	0.25 AB	1.57 AC	35.9 AC	48.9 AB	2.32 AC	0.67 AC	0.58 A
		Gavdos	10.0 bc	0.22 AB	1.96 AB	34.1 AC	49.9 AB	2.45 AC	0.73 AB	0.56 A
		Lambada	10.1 bc	0.23 AB	1.42 C	35.0 AC	49.6 AB	2.36 AC	0.67 AC	0.61 A
		APSC4	10.1 bc	0.23 AB	1.88 AC	38.8 AC	45.7 B	2.08 C	0.70 AC	0.56 A
		APSC3	9.90 bc	0.21 AB	1.67 AC	38.9 AB	46.0 AB	2.16 AC	0.66 AC	0.58 A
		Cicerone	10.0 bc	0.22 AB	1.63 AC	37.0 AC	47.6 AB	2.29 AC	0.68 AC	0.59 A
		Vulcano	10.7 a	0.27 AB	1.64 AC	33.7 AC	50.2 AB	2.39 AC	0.65 BC	0.52 A
		Sultano	10.2 b	0.22 AB	1.71 AC	39.0 A	45.5 B	2.11 BC	0.71 AC	0.61 A
		Flamenco	9.90 bc	0.22 AB	1.49 AC	32.7 AC	52.1 AB	2.46 AB	0.58 C	0.54 A
	desi type, black	Nero	11.0 a	0.28 A	1.45 BC	29.8 C	53.5 A	2.64 A	0.70 AC	0.61 A

a All data are expressed as the average of 3 replicates of the percentage value of each single fatty acid compared to the total fat content.

b Within each crop, different lowercase letters indicate significant differences among genotypes according to ANOVA and Tukey’s multiple comparison test. Capital letters denote significant differences identified by Dunn’s post hoc test applied to nonparametric data (following Kruskal–Wallis test).

### Bioactive Compounds and Phytochemicals

#### Carotenoids

The highest TCC was observed in field peas, with an average of 5.0 mg of β-carotene equivalents 100 g^–1^ d.w., significantly higher than chickpeas, which exhibited an average of 2.2 mg of β-carotene equivalents 100 g^–1^ d.w. (−58%) (Table 5). These differences were strongly influenced by seed pigmentation. Yellow field pea cvs showed limited variation, ranging from 1.8 (Navarro) to 2.4 mg 100 g^–1^ d.w. (RGT Lapony), while green cvs exhibited much higher values, from 5.7 mg 100 g^–1^ (Verbal) to 8.6 mg 100 g^–1^ (Bluemoon), +70% more than yellow ones.

**Table 5:** Total Phenolic Content (TPC), Total Flavonoid Content (TFC), Soluble Phenolic Acid (SPA), Cell-Wall Bound Phenolic Acid (CWBPA) Content, Total Carotenoid Content (TCC), and Antioxidant Capacity (AC) Measured with Different Assays (FRAP and ABTS)^a^

**crop**	**qualitative trait**	**cultivar**	**TPC (mg CAE g** ^**–1**^)	**TFC (mg kg** ^**–1**^)	**SPA (mg kg** ^**–1**^)	**CWBPA (mg kg** ^**–1**^)	**TCC (mg β-carotene 100 g** ^**–1**^)	**AC _**FRAP**_ (mmol TE kg** ^**–1**^)	**AC**_**ABTS**_ (**mmol TE kg** ^**–1**^)
field pea	green	Standal	2.72 bcde	37.19 A	15.99 ef	13.33 AB	6.37 c	4.4 cd	22.6 bc
		Verbal	2.81 bcde	106.18 A	65.79 b	39.50 AB	5.61 d	5.7 abc	24.2 abc
		Faquir	2.61 cde	51.14 A	19.84 de	16.51 AB	7.09 b	5.1 cd	22.3 c
		LS Envergure	2.25 e	48.95 A	25.47 d	17.54 AB	7.09 b	5.0 cd	23.0 bc
		Paddle	2.62 cde	47.77 A	19.53 de	18.38 AB	7.07 b	5.2 bcd	25.3 ab
		Aviron	2.53 de	39.61 A	15.49 ef	10.23 B	8.15 a	5.0 cd	23.4 bc
		Bluemoon	2.44 e	42.87 A	17.46 def	15.26 AB	8.55 a	5.1 cd	23.3 bc
	yellow	Astronaute	3.33 abc	121.4 A	19.43 def	33.13 AB	2.33 e	6.5 ab	24.4 abc
		Navarro	3.68 a	92.56 A	55.24 c	38.38 AB	1.84 e	5.6 abc	23.8 bc
		Angelus	3.28 bcde	154.20 A	74.37 a	52.41 A	1.86 e	6.7 a	26.8 a
		RGT Lapony	3.43 ab	49.11 A	14.18 f	8.74 B	2.35 e	4.2 d	22.0 c
		Enduro	2.84 bcde	42.81 A	16.33 ef	21.27 AB	2.04 e	5.6 abc	24.0 abc
									
chickpea	kabuli type, beige	Alamo	1.44 c	33.45 A	15.75 A	11.94 a	1.80 AB	3.3 AB	22.3 A
		Pascià	1.53 c	30.44 A	12.41 A	10.46 ab	1.91 AB	3.3 AB	21.0 A
		Gavdos	1.92 ab	28.93 A	11.30 A	8.37 bcd	2.09 AB	3.4 AB	21.7 A
		Lambada	1.61 c	29.29 A	13.65 A	8.30 cd	1.97 AB	3.1 AB	22.2 A
		APSC4	1.66 bc	25.74 A	17.70 A	8.30 cd	1.91 AB	3.2 AB	21.8 A
		APSC3	1.68 bc	26.20 A	10.95 A	7.32 d	1.89 AB	3.1 AB	22.6 A
		Cicerone	1.73 bc	29.87 A	16.06 A	12.04 a	2.12 AB	3.1 AB	22.7 A
		Vulcano	1.74 bc	28.35 A	13.14 A	7.86 cd	1.98 AB	3.0 B	27.6 A
		Sultano	1.73 bc	28.55 A	14.97 A	10.16 abc	2.02 AB	3.3 AB	21.2 A
		Flamenco	2.04 a	25.76 A	12.06 A	8.37 bcd	1.69 B	3.2 AB	22.1 A
	desi type, black	Nero	1.58 c	37.18 A	9.47 A	10.00 abcd	4.33 A	20.7 A	27.6 A

a All data are expressed as the average of 3 replicates and expressed on d.w. of the sample. Within each crop, different lowercase letters indicate significant differences among genotypes according to ANOVA and Tukey’s multiple comparison test. Capital letters denote significant differences identified by Dunn’s posthoc test applied to nonparametric data (following Kruskal–Wallis test).

Among chickpeas, kabuli cvs had a lower average TCC (1.9 mg of β-carotene equivalents 100 g^–1^ d.w.), which is in line with the findings of Consumi et al. (2022)^42^ (1.3 mg 100 g^–1^), whereas the desi cv reached 4.3 mg β-carotene equivalents 100 g^–1^ d.w. (+55%). From a technological perspective, these carotenoids influence the final product pigmentation and could increase after thermal treatments such as steaming or water heating.^43^ Furthermore, storage conditions can have a negative impact on their stability due to the oxygen radical production from fatty aciddegradation, which is primarily mediated by lipoxygenases.^44^

#### Phenolic Content

The TPC was significantly higher in field peas than in chickpeas (Table 5), with average values of 2.9 and 1.7 mg CAE g^–1^ d.w., respectively (−42%). TPC in yellow peas (3.3 mg CAE g^–1^ d.w.) was significantly higher (+26%) than that of green pea ones (2.5 mg CAE g^–1^ d.w.), in accordance with those reported by Han and Baik (2008),^45^ who estimated values of 2.5 and 1.2 mg g^–1^ d.w. for yellow and green peas, respectively. The highest TPC was observed in the Navarro cv (3.7 mg CAE g^–1^ d.w.), while the Bluemoon and LS Envergure cvs showed the lowest values. In chickpea samples, Flamenco and Gavdos cvs exhibited the highest TPC (2.0 and 1.9 mg CAE g^–1^ d.w., respectively). Conversely, the Alamo, Pascià, Lambada, and Nero cvs displayed values that were significantly lower than the average concentration.

Regarding SPAs and CWBPAs, field pea genotypes exhibited significantly higher concentrations than chickpeas (+64% and +71%, respectively). In field pea genotypes, SPAs were generally 20% more abundant than CWBPAs, with yellow cvs showing higher average concentrations than the green ones for both classes of compounds (+48% and +51%, respectively). Angelus (yellow) and Verbal (green) showed the highest content of CWBPAs compared to the average (+62% and +56%, respectively), whereas RGT Lapony (yellow) and Bluemoon (green) showed the lowest ones. In chickpea genotypes, APSC4 and Cicerone cvs showed the highest SPA and CWBPA contents (+32% and +27%, respectively), whereas Nero and APSC3 presented the lowest ones (−15% and −23%, respectively).

#### Flavonoid Content and Profile

Total flavonoid content (TFC) followed a trend similar to TPC, with field peas exhibiting significantly higher values than chickpeas (69.5 and 29.3 mg kg^–1^, respectively) (Table 6). However, Dunn’s test revealed no significant differences between the samples. All the genotypes were revealed to contain catechin, epicatechin, rutin (quercetin 3*-O-*rutinoside), hyperoside (quercetin 3*-O-*galactoside), quercitrin, apigenin 7*-O-*glucoside, and myricetin (Table 6), with catechin, epicatechin, and apigenin 7*-O-*glucoside being the most representative flavonoids in field pea, while epicatechin in chickpea. Catechin content was substantially higher in field peas (28.0 mg kg^–1^ d.w.) compared to chickpeas (2.6 mg kg^–1^ d.w.), while chickpeas had greater levels of hyperoside and quercitrin. As reported by Quintero-Soto et al. (2018),^46^ rutin was poorly present in both types of chickpeas.

**Table 6:** Flavonoid Profile of Field Pea and Chickpea Cultivars^a^

crop	qualitative trait	cultivar	catechin	epicatechin	rutin	hyperoside	quercitrin	apigenin 7*-O-*glucoside	myricetin
field pea	green	Standal	21.7 A	5.69 c	5.07 ab	0.11 A	0.53 A	3.45 A	0.68 c
		Verbal	34.5 A	45.6 b	4.17 bc	ND	1.66 A	17.9 A	2.33 b
		Faquir	30.1 A	7.23 c	4.30 bc	0.54 A	0.61 A	7.42 A	0.96 c
		LS Envergure	26.4 A	9.15 c	4.77 abc	0.40 A	0.78 A	6.50 A	0.92 c
		Paddle	26.6 A	11.3 c	4.38 bc	0.27 A	0.53 A	3.71 A	0.93 c
		Aviron	20.8 A	11.4 c	3.82 c	0.28 A	0.50 A	2.38 A	0.43 c
		Bluemoon	22.1 A	13.1 c	4.10 bc	0.23 A	0.43 A	2.40 A	0.53 c
	yellow	Astronaute	32.6 A	62.6 a	2.56 d	0.28 A	1.75 A	19.7 A	1.87 b
		Navarro	20.8 A	46.6 b	2.24 d	0.50 A	2.58 A	19.9 A	ND
		Angelus	47.0 A	66.1 a	5.67 a	1.04 A	2.41 A	26.1 A	5.76 a
		RGT Lapony	31.6 A	8.31 c	5.02 ab	0.63 A	0.38 A	3.15 A	ND
		Enduro	21.6 A	11.1 c	4.65 bc	1.07 A	0.77 A	2.88 A	0.78 c
									
chickpea	kabuli type, beige	Alamo	3.66 b	21.0 a	0.76 A	1.87 A	4.11 A	0.60 A	1.43 ab
		Pascià	1.83 fg	20.5 ab	0.61 A	1.43 A	3.66 A	0.61 A	1.68 a
		Gavdos	2.20 ef	17.5 cd	0.72 A	1.38 A	5.03 A	0.76 A	1.28 bc
		Lambada	1.72 g	18.4 cd	0.62 A	1.35 A	4.92 A	0.80 A	1.33 bc
		APSC4	2.47 cde	14.0 e	0.66 A	1.76 A	4.91 A	0.64 A	1.33 bc
		APSC3	2.84 c	15.3 e	0.27 A	1.55 A	4.01 A	0.72 A	1.43 ab
		Cicerone	2.41 de	20.8 ab	0.34 A	1.44 A	3.55 A	0.34 A	0.88 def
		Vulcano	3.73 b	17.4 d	0.58 A	1.33 A	3.95 A	0.49 A	0.77 ef
		Sultano	2.64 cd	19.2 bc	0.46 A	1.30 A	3.12 A	0.73 A	0.98 de
		Flamenco	1.23 h	15.4 e	0.22 A	3.31 A	4.16 A	0.37 A	1.06 cd
	desi type, black	Nero	4.20 a	21.8 a	0.68 A	6.81 A	1.79 A	0.82 A	0.67 f

a All data are expressed as the average of 3 replicates and expressed as mg kg^–1^ d.w. ND: not detected. Within each crop, different lowercase letters indicate significant differences among genotypes according to ANOVA and Tukey’s multiple comparison test. Capital letters denote significant differences identified by Dunn’s post hoc test applied to nonparametric data (following Kruskal–Wallis test).

Dunn’s test revealed no significant difference between field pea cvs in catechin, hyperoside, quercitrin, and apigenin 7*-O-*glucoside content. However, Angelus showed the highest levels of several key compounds, including epicatechin (66.1 mg kg^–1^ d.w.) and rutin (5.7 mg kg^–1^ d.w.). In contrast, Standal and Aviron exhibited markedly lower levels of catechin and epicatechin. No significant differences were found in rutin, hyperoside, quercitrin, and apigenin 7*-O-*glucoside content between chickpea cvs, while Nero showed the highest catechin (4.2 mg kg^–1^ d.w.) content compared to the kabuli cvs.

#### Antioxidant Capacity (AC)

In vitro antioxidant capacity (AC) assays showed that field pea cultivars exhibited higher AC than kabuli-type chickpeas, with average FRAP values of 5.3 and 3.2 mmol TE kg^–1^ d.w., respectively. The ABTS assay confirmed this trend, revealing a 5% higher AC in field peas compared to kabuli chickpeas. The only exception was the desi cultivar Nero, which displayed markedly elevated AC relative to all samples: +75% in FRAP and +15% in ABTS compared to the field pea cultivars. This observation is consistent with findings by Xu et al. (2007),^47^ who reported that darker seed coats are associated with higher antioxidant capacity due to increased levels of anthocyanins and condensed tannins. Significant differences in AC were observed among field pea cultivars with cv. Angelus showing the highest values (6.7 and 26.8 mmol TE kg^–1^ in FRAP and ABTS, respectively). In contrast, no significant differences in ABTS-derived AC were detected among kabuli chickpea cultivars, in agreement with results reported by Kaur et al. (2019).^48^

#### Multivariate Analysis of Bioactive Compounds and Phytochemicals

To explore the variability in bioactive compound content among genotypes and identify cvs with enhanced functional potential, the results of TPC, TFC, SPA, CWBPA, TCC, AC_FRAP_, and AC_ABTS_ were subjected to principal component analysis (PCA) and hierarchical clustering on principal components (HCPC). This multivariate approach enables the identification of cvs particularly rich in phenolics and carotenoids, which could add nutritional value to wholemeal flours and their derived products. The growing demand for health-promoting ingredients in everyday foods, such as pasta and snacks, further underscores the importance of this screening. Bioactive compounds, particularly phenolics, contribute to AC through multiple mechanisms, including metal chelation, free radical scavenging, and inhibition of lipid oxidation pathways.^49,50^ In field peas, clustering did not align strictly with the seed coat color (Figure 1). Cluster 1 included both yellow (RGT Lapony and Enduro) and green (Bluemoon) cvs negatively associated with TPC (*p-*value = 0.03), AC_FRAP_ (*p-*value = 0.01), SPA (*p-*value = 0.008), CWBPA (*p-*value = 0.002), and TFC (*p-*value = 0.003). In contrast, the green cv Verbal is grouped with yellow cvs in cluster 2 due to the positive influence of the same variables. Angelus emerged as the most distinct genotype, characterized by elevated levels of TFC, SPA, and CWBPA, which contribute to its strong antioxidant potential (AC_FRAP_ and AC_ABTS_), making it a promising candidate for the development of nutritionally enhanced bakery products.

**Figure 1:**
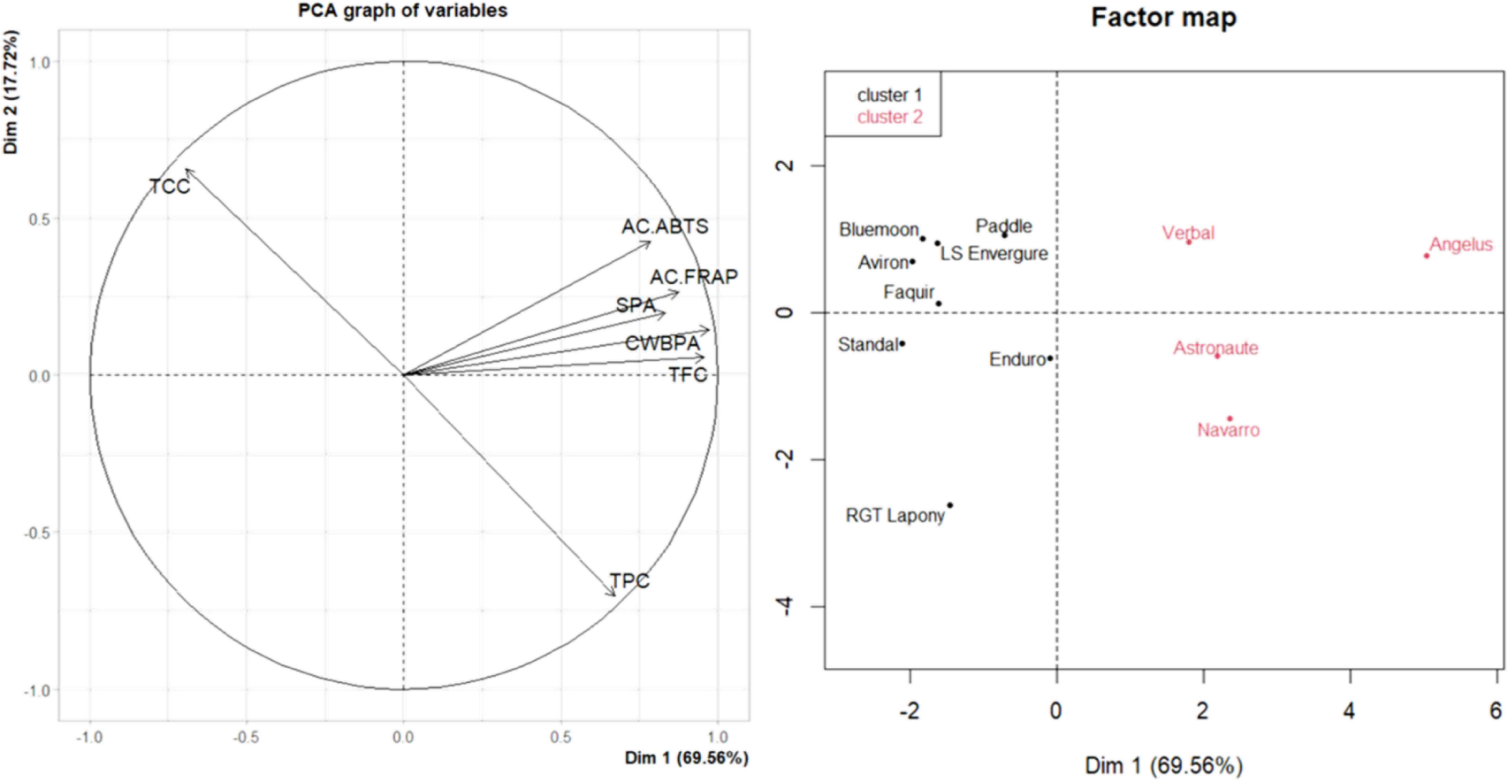
PCA graph of variables (PC1 vs PC2) and HCPC’s factor map of field pea flours. Green cvs: Standal, Verbal, Faquir, LS Envergure, Paddle, Aviron, and Bluemoon. Yellow cvs: Astronaute, Navarro, Angelus, RGT Lapony, and Enduro. TPC, total phenolic content; TFC, total flavonoid content; SPA, soluble phenolic acid; CWBPA, cell-wall bound phenolic acid content; TCC, total carotenoid content; AC_FRAP_ and AC_ABTS_, antioxidant capacity measured with different assays.

Three clusters were identified considering the chickpea clusters (Figure 2). Kabuli cvs were distributed into two groups based primarily on CWBPA content. Cluster 1 (black) included Sultano, APSC4, APSC3, Lambada, Vulcano, Flamenco, and Gavdos, and it was characterized by lower TFC (*p-*value = 0.01) and CWBPA (*p-*value = 0.008). Cluster 2 (red), which included Alamo, Cicerone, and Pascià, showed higher CWBPA concentrations (*p-*value = 0.009). The desi cv Nero formed a distinct third cluster (green), with notably higher levels of carotenoids and phenolics, consistent with previous studies conducted in different environments.^51^ Variables such as TCC, TFC, AC_FRAP_, and AC_ABTS_ significantly contributed to the clustering pattern (*p-*value = 0.002), highlighting Nero’s potential as a valuable raw material for functional ingredient development.

**Figure 2:**
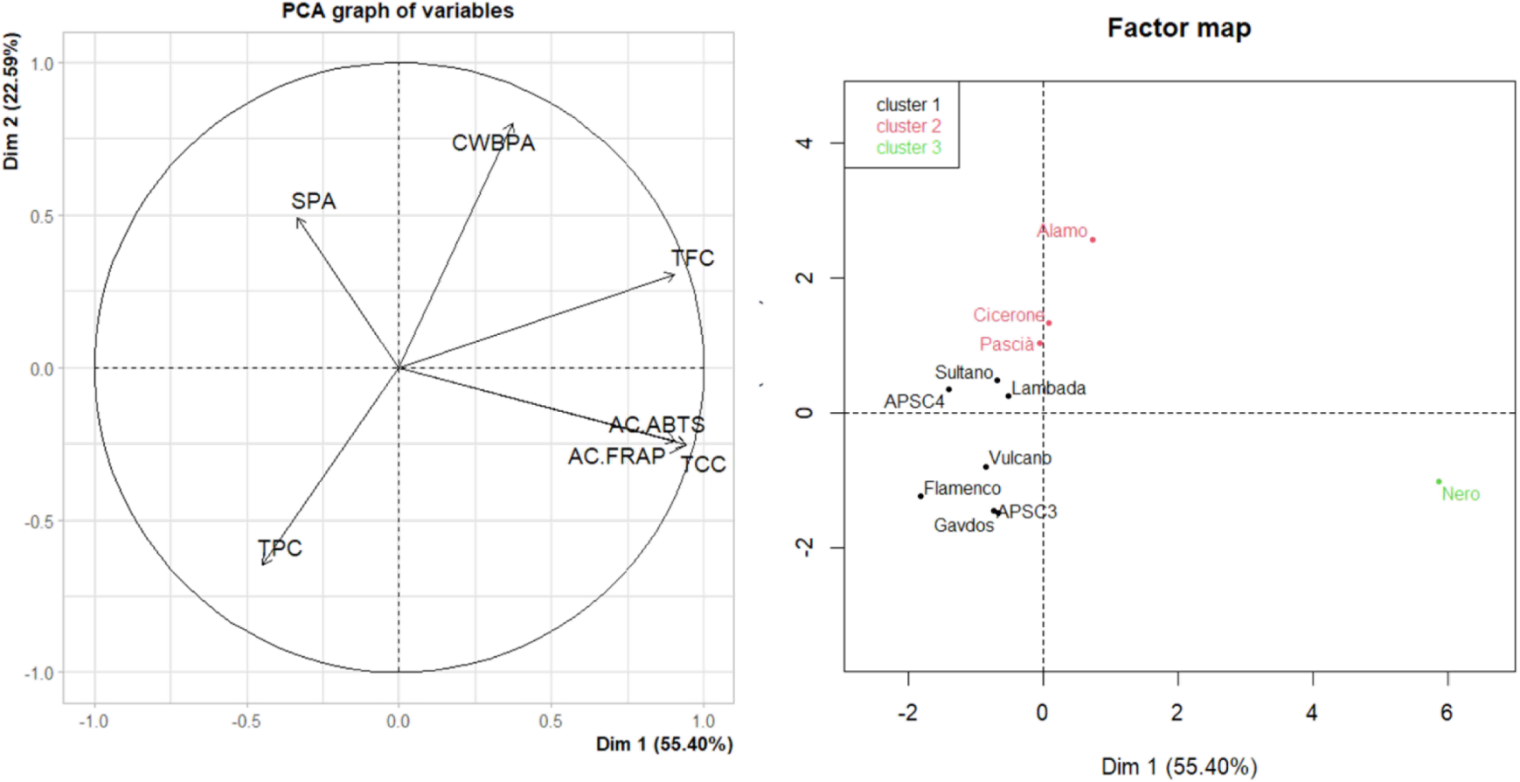
PCA graph of variables (PC1 vs PC2) and HCPC’s factor map of chickpea flours. Kabuli cvs: Alamo, Pascià, Gavdos, Lambada, APSC4, APSC3, Cicerone, Vulcano, Flamenco, and Sultano. Desi cv: Nero. TPC, total phenolic content; TFC, total flavonoid content; SPA, soluble phenolic acid; CWBPA, cell-wall bound phenolic acid content; TCC, total carotenoid content; AC_FRAP_ and AC_ABTS_, antioxidant capacity measured with different assays.

In conclusion, this study aimed to characterize the nutritional and phytochemical profiles of multiple chickpea and field pea crops grown under identical agronomic conditions, with the goal of identifying promising raw materials for the development of functional and plant-based food products. The findings confirm that both legume species offer valuable sources of macronutrients and bioactive compounds, though with distinct composition traits: chickpeas exhibited higher protein and lipid contents, whereas field peas were richer in starch and TDF. These differences suggest cultivar-specific applications; for example, field peas like Bluemoon, with high starch content, may be optimal for extruded products, while chickpeas, particularly the desi genotype Nero, stand out for their antioxidant capacity and TDF content. Among field peas, Angelus cv was particularly rich in phenolic compounds, contributing to its superior antioxidant potential. In contrast, most kabuli chickpea cvs showed compositional similarities, with Nero (desi type) emerging as a unique source of functional compounds. These insights support the more targeted selection of legume flours for product development, potentially aligning breeding and processing strategies with the nutritional and functional demands of the modern food industry. A major strength of this study lies in its systematic comparison of genotypes under uniform environmental conditions, allowing for clear attribution of differences to genetic variation rather than environmental effects. However, the lack of multienvironment trials limits the generalizability of the findings. Moreover, the absence of sensory or technological evaluations in real food matrices prevents direct translation into product formulations. Future studies should expand the varietal screening to different growing environments and explore how these compositions translate into functional, sensory, and shelf-life properties of legume-based food products. Integration of genotype data with technological performance could enhance CV selection for specific applications in extrusion, baking, or fermentation processes. These efforts will help develop more sustainable, nutritious, and appealing plant-based foods that meet consumer and industry needs.

## Supplementary Material



## Notes

Table S1. The field pea and chickpea cultivars compared in the study and their agronomic traits; Table S2. Monthly rainfall and average daily temperature at the experimental site, during the cultivation of field pea (November–June) and chickpea (April–August); Table S3. Results of the Shapiro–Wilk normality test for the analyzed parameters in field pea and chickpea samples. The table reports the Shapiro–Wilk statistic (*W*), the corresponding *p*-value, and the outcome of the normality test at α = 0.05. Parameters with *p* < 0.05 were considered to deviate significantly from a normal distribution (“No”), indicating nonparametric data behavior. Parameters with *p* ≥ 0.05 passed the normality assumption (“Yes”) and were thus considered suitable for parametric statistical analysis. Symbols indicate the level of significance (*p* < 0.05 = *, <0.01 = **, <0.001 = ***, <0.0001 = ****, ns = not significant); Figure S1. Concentration of the main nonessential amino acids (NEAAs) and essential amino acids (EAAs) found in field pea (dark gray) and chickpea (light gray) genotypes. Analysis was carried out on a representative merged sample of field-replicated plots. Asx, aspartic acid + asparagine; Glx, glutamic acid + glutamine; Ser, serine; Gly, glycine; Ala, alanine; Arg, arginine; Pro, proline; Cys, cysteine + cystine; Tyr, tyrosine; Phe, phenylalanine; Ile, isoleucine; His, histidine; Leu, leucine; Lys, lysine; Met, methionine; Thr, threonine; Trp, tryptophan; Val, valine; Figure S2. Total content of essential amino acids (EAAs, dark blue) and nonessential amino acids (NEAAs, light blue) in field pea and chickpea genotypes. Analysis was carried out on a representative merged sample of field-replicated plots (PDF)

## Abbreviations

CV: coefficient of variation
cv/cvs: cultivar/cultivars
GC–FID: gas chromatography–flame ionization detector
HPLC–DAD: high performance liquid chromatography–diode array detector
HPLC–FLD: high performance liquid chromatography–fluorescence detector
HPLC–UV: high performance liquid chromatography–ultraviolet detector
FAME: fatty acid methyl ester
TPC: total phenolic content
TFC: total flavonoid content
CWBPA: cell-wall bound phenolic acid
SPA: soluble phenolic acid
TDF: total dietary fiber
CAE: catechin equivalents
TE: Trolox equivalents
Rt: retention time
FRAP: ferric reducing antioxidant power
ABTS: [2,2’-azino-*bis*(3-ethylbenzothiazoline-6-sulfonic acid)]
QUENCHER: QUick, Easy, New, CHEap and Reproducible
EAA: essential amino acid
NEAA: nonessential amino acid
AC: antioxidant capacity
ANOVA: analysis of variance
PCA: principal component analysis
HCPC: hierarchical clustering on principal component analysis
f.w.: fresh weight
d.w.: dry weight
EFSA: European Food Safety Authority
BSAE: bovine serum albumin equivalent
MUFA: monounsaturated fatty acid
PUFA: polyunsaturated fatty acid
LA: linoleic acid
ALA: α-linolenic acid
SFA: saturated fatty acid
TAA: total amino acid content
LoQ: limit of quantification
DRV: dietary reference values
NDA: The Panel on Nutrition, Novel Foods and Food Allergens of EFSA
Asx: aspartic acid + asparagine
Glx: glutamic acid + glutamine
Ser: serine
Gly: glycine
Ala: alanine
Arg: arginine
Pro: proline
Cys: cysteine + cystine
Tyr: tyrosine
Phe: phenylalanine
Ile: isoleucine
His: histidine
Leu: leucine
Lys: lysine
Met: methionine
Thr: threonine
TKW: thousand kernel weight
TW: test weight
Trp: tryptophan
Val: valine
