# Supplementary material for: Nutritional Composition and Content of Bioactive Compounds in Field Pea and Chickpea Varieties as Functional Raw Material for the Food Supply Chain
Source: ACS Food Sci Technol. 2025 Dec 3;5(12):4620–31. doi: 10.1021/acsfoodscitech.5c00941 (PMC12724375; doi:10.1021/acsfoodscitech.5c00941)
Supplement: Supplementary file 1 [file fs5c00941_si_001.pdf]

Supporting information description

Table S1.

The field pea and chickpea cultivars compared in the study and their agronomic traits.

crop	cultivar	qualitative traits	cicle	seed company	cultivar
field pea	Standal	green color	medium-early	Mas seed s.r.., San Pietro di Morubio (VR), Italy	commercial
	Verbal	green color	medium	Mas seed s.r.., San Pietro di Morubio (VR), Italy	commercial
	Faquir	green color	medium-late	Apsovsementi S.p.a., Voghera (PV), Italy	commercial
	LS Envergure	green color	medium	Mas seed s.r.., San Pietro di Morubio (VR), Italy	commercial
	Paddle	green color	medium-late	Società Italiana Sementi, S. Lazzaro di Sabena (BO), Italy	commercial
	Aviron	green color	medium	Società Produttori Sementi S.p.A., Argelato (BO), Italy	commercial
	Bluemoon	green color	medium	Apsovsementi S.p.a., Voghera (PV), Italy	commercial
	Astronaute	yellow color	medium	Società Produttori Sementi S.p.A., Argelato (BO), Italy	commercial
	Navarro	yellow color	medium-early	Società Italiana Sementi, S. Lazzaro di Sabena (BO), Italy	commercial
	Angelus	yellow color	medium	Adaglio Sementi S.r.l., Oviglio (AL), Italy	commercial
	RGT Lapony	yellow color	medium-late	Società Italiana Sementi, S. Lazzaro di Sabena (BO), Italy	commercial
	Enduro	yellow color	medium	Adaglio Sementi S.r.l., Oviglio (AL), Italy	commercial
chickpea	Alamo	kabuli, wrinkled beige pericarp	medium-early	Apsovsementi S.p.a., Voghera (PV), Italy	commercial
	Pascià	kabuli, wrinkled beige pericarp	medium-early	Società Produttori Sementi S.p.A., Argelato (BO), Italy	commercial
	Gavdos	kabuli, wrinkled pericarp	early	Adaglio Sementi S.r.l., Oviglio (AL), Italy	commercial
	Lambada	kabuli, wrinkled pericarp	medium-early	Novasem s.r.l., Genola (CN), Italy	commercial
	APSC4	kabuli, wrinkled pericarp	medium-early	Apsovsementi S.p.a., Voghera (PV), Italy	pre-commercial
	APSC3	kabuli, wrinkled pericarp	medium-early	Apsovsementi S.p.a., Voghera (PV), Italy	pre-commercial
	Cicerone	kabuli, wrinkled pericarp	medium	Società Italiana Sementi, S. Lazzaro di Sabena (BO), Italy	commercial
	Vulcano	kabuli, wrinkled pericarp	early	Società Italiana Sementi, S. Lazzaro di Sabena (BO), Italy	commercial
	Flamenco	kabuli, wrinkled pericarp	early	Novasem s.r.l., Genola (CN), Italy	commercial
	Sultano	kabuli, smooth beige pericarp	medium-early	Società Produttori Sementi S.p.A., Argelato (BO) – Italy	commercial
	Nero	desi, wrinkled black pericarp	medium	-	local ecotype

Table S2.

Monthly rainfall and average daily temperature at the experimental site, during the cultivation of field pea (November – June) and chickpea (April – August).

year	month	cumulative rainfall (mm)	average daily temperature (°C)
2021	November	164	6.5
	December	8	0.5
2022	January	0	1.0
	February	1	4.6
	March	15	6.9
	April	27	12.3
	May	76	19.2
	June	19	23.6
	July	58	25.9
	August	71	23.6
field pea	growing season	309	9.3
chickpea	growing season	250	20.9

Source: Rete Agrometeorologica del Piemonte - Regione Piemonte - Assessorato Agricoltura - Settore Fitosanitario. sezione di Agrometeorologia. Weather station located in Polonghera, 2 km far from the experimental site.

Table S3. Results of the Shapiro-Wilk normality test for the analyzed parameters in field pea and chickpea samples. The table reports the Shapiro-Wilk statistic (W), the corresponding p-value, and the outcome of the normality test at α = 0.05. Parameters with $p < 0.05$ were considered to deviate significantly from a normal distribution ("No"), indicating non-parametric data behavior. Parameters with $p \geq 0.05$ passed the normality assumption ("Yes") and were thus considered suitable for parametric statistical analysis. Symbols indicate the level of significance (p < 0.05 = *, < 0.01 = **, < 0.001 = ***, < 0.0001 = ****, ns = not significant).

	Field pea				Chickpea			
**Parameter**	**W**	***p*-value**	**α =0.05?**	***p*-value summary**	**W**	***p*-value**	**α =0.05?**	***p*-value summary**
**grain yield**	0.901	0.0037	No	**	0.955	0.184	Yes	ns
**TKW**	0.917	0.0489	No	*	0.942	0.213	Yes	ns
**TW**	0.938	0.1439	Yes	ns	0.974	0.796	Yes	ns
**moisture**	0.911	0.0068	No	**	0.944	0.089	Yes	ns
**starch**	0.895	0.0025	No	**	0.830	<0.0001	No	***
**total dietary fiber**	0.961	0.4488	Yes	ns	0.692	<0.0001	No	****
**lipid**	0.972	0.7093	Yes	ns	0.957	0.431	Yes	ns
**protein**	0.977	0.6448	Yes	ns	0.931	0.116	Yes	ns
**ash**	0.988	0.9899	Yes	ns	0.942	0.217	Yes	ns
**albumins**	0.982	0.8665	Yes	ns	0.967	0.498	Yes	ns
**globulins**	0.865	0.0023	No	**	0.963	0.446	Yes	ns
**glutelins**	0.968	0.5941	Yes	ns	0.914	0.058	Yes	ns
**prolamins**	0.938	0.1438	Yes	ns	0.920	0.0758	Yes	ns
**C16:0**	0.923	0.0158	No	*	0.937	0.056	Yes	ns
**C16:1**	0.807	<0.0001	No	****	0.754	<0.0001	No	****
**C18:0**	0.878	0.0009	No	***	0.430	<0.0001	No	****
**C18:1n9cis**	0.961	0.2250	Yes	ns	0.928	0.030	No	*
**C18:2n6cis**	0.941	0.0533	Yes	ns	0.924	0.024	No	*
**C18:3n3**	0.952	0.1249	Yes	ns	0.904	0.007	No	**
**C20:0**	0.946	0.0784	Yes	ns	0.416	<0.0001	No	****
**C20:1**	0.968	0.3827	Yes	ns	0.590	<0.0001	No	****
**TPC**	0.617	<0.0001	No	****	0.958	0.228	Yes	ns
**TFC**	0.775	0.0001	No	***	0.895	0.0232	No	*
**SPA**	0.745	<0.0001	No	****	0.902	0.006	No	**
**CWBPA**	0.869	0.0007	No	***	0.943	0.083	Yes	ns
**TCC**	0.817	<0.0001	No	****	0.516	<0.0001	No	****
**AC_{FRAP}**	0.957	0.1671	Yes	ns	0.899	0.005	No	**
**AC_{ABTS}**	0.963	0.2732	Yes	ns	0.363	<0.0001	No	****
**catechin**	0.871	0.0055	No	**	0.948	0.287	Yes	ns
**epicatechin**	0.747	<0.0001	No	****	0.938	0.179	Yes	ns
**rutin**	0.919	0.0567	Yes	ns	0.887	0.017	No	*
**hyperoside**	0.870	0.0078	No	**	0.557	<0.0001	No	****
**quercitrin**	0.785	0.0002	No	***	0.911	0.049	No	*
**apigenin 7-*O*-glucoside**	0.783	0.0002	No	***	0.910	0.048	No	*
**myricetin**	0.659	<0.0001	No	****	0.963	0.551	Yes	ns

Figure S1.

Concentration of the main non-essential amino acids (NEAAs) and essential amino acids (EAAs) found in field pea (dark gray) and chickpea (light gray) genotypes.

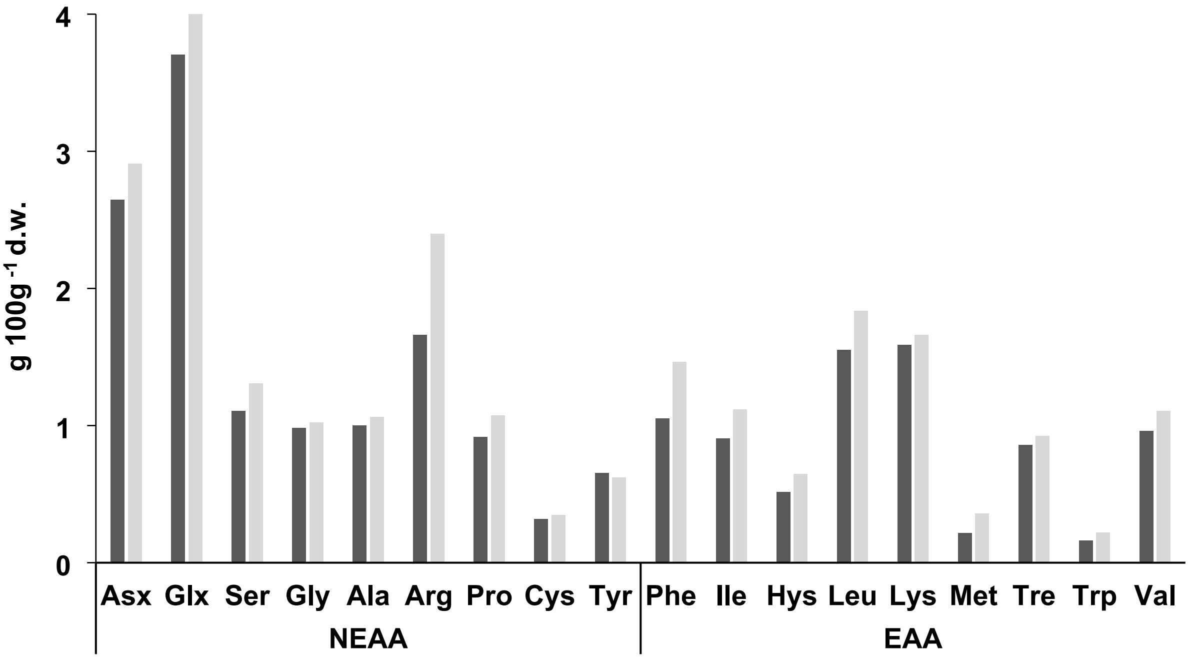

Analysis was carried out on a representative merge sample of field replicated plots. Asx, aspartic acid + asparagine; Glx, glutamic acid + glutamine; Ser, serine; Gly, glycine; Ala, alanine; Arg, arginine; Pro, proline; Cys, cysteine + cystine; Tyr, tyrosine; Phe, phenylalanine; Ile, isoleucine; His, histidine; Leu, leucine; Lys, lysine; Met, methionine; Thr, threonine; Trp, tryptophan; Val, valine.

Figure S2.

Total content of essential amino acids (EAAs, dark blue) and non-essential amino acids (NEAAs, light blue) in field pea and chickpea genotypes.

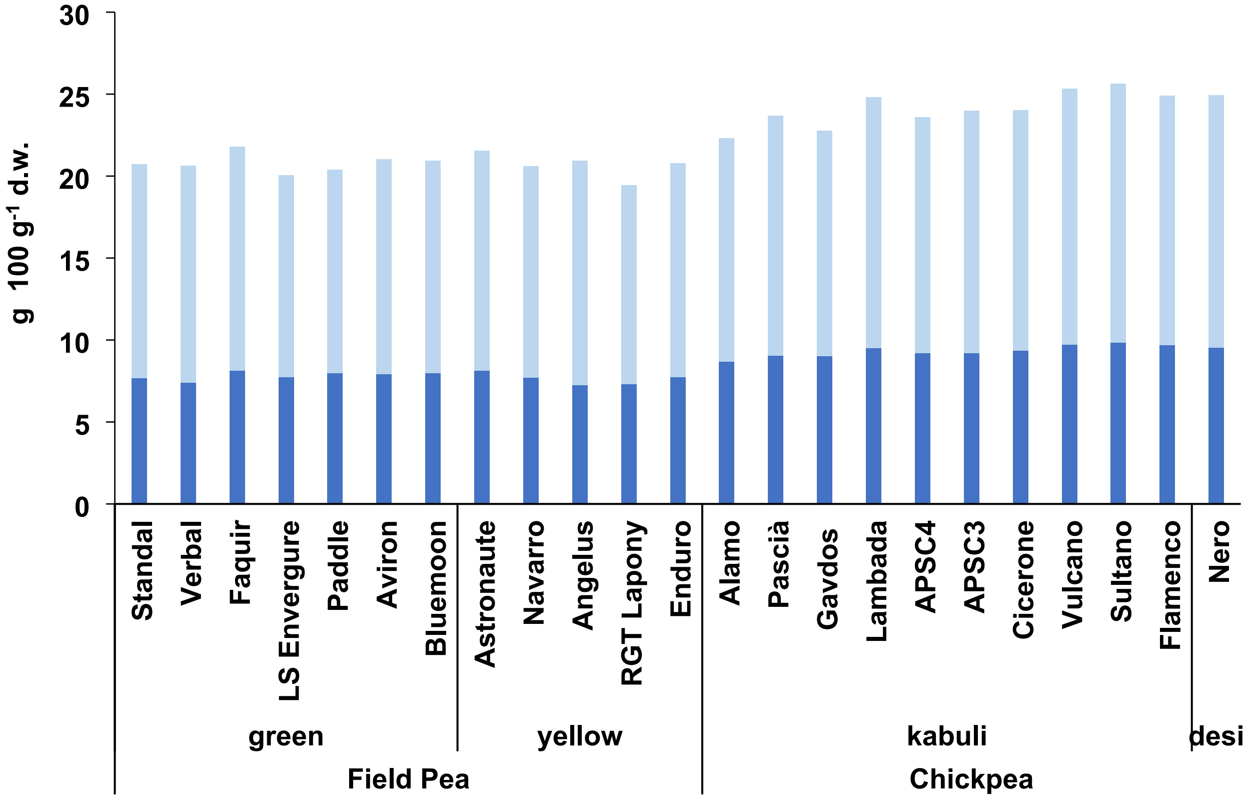

Analysis was carried out on a representative merge sample of field replicated plots.
